# Supplementary material for: The Systems Biology Research Tool: evolvable open-source software
Source: BMC Syst Biol. 2008 Jun 29;2:55. doi: 10.1186/1752-0509-2-55 (PMC2446383; doi:10.1186/1752-0509-2-55)
Supplement: Additional file 1 — SBRT Archive. An archive of the current version of the Systems Biology Research Tool. [file 1752-0509-2-55-S1.zip › sbrt-1.4.0/doc/developers_guide/api/sbrt/ext/mma/algebra/package-summary.html]

sbrt.ext.mma.algebra


|  |  |  |
| --- | --- | --- |
| **Overview** \| **Package** \| Class \| **Use** \| **Tree** \| **Deprecated** \| **Index** \| **Help** | |  |
| **PREV PACKAGE**   **NEXT PACKAGE** | **FRAMES**    **NO FRAMES**     **All Classes** |  |


---

## Package sbrt.ext.mma.algebra

Provides classes that allow communication with Mathematica for the
purposes of algebra.

**See:**
  
          **Description**

| **Class Summary** | |
| --- | --- |
| **MathematicaSolver<V>** | This class is used to solve systems of linear equations using Mathematica. |
| **MMaExprInterpretter** | This class is intended for use solely by the class `MathematicaSolver`. |

## Package sbrt.ext.mma.algebra Description

Provides classes that allow communication with Mathematica for the
purposes of algebra.

---


|  |  |  |
| --- | --- | --- |
| **Overview** \| **Package** \| Class \| **Use** \| **Tree** \| **Deprecated** \| **Index** \| **Help** | |  |
| **PREV PACKAGE**   **NEXT PACKAGE** | **FRAMES**    **NO FRAMES**     **All Classes** |  |


---
